# NFC-Enabled Dual-Channel Flexible Printed Sensor Tag

**DOI:** 10.3390/s23156765

**Published:** 2023-07-28

**Authors:** Jonghyun Choi, Ian Visagie, Yi Chen, Robert Abbel, Kate Parker

**Affiliations:** 1 The New Zealand Institute for Plant and Food Research Ltd., Private Bag 3230, Waikato Mail Centre, Hamilton 3240, New Zealand; ian.visagie@plantandfood.co.nz; 2 Scion, New Zealand Forest Research Institute Ltd., Tītokorangi Drive, Private Bag 3020, Rotorua 3046, New Zealand; robert.abbel@scionresearch.com (R.A.); kate.parker@scionresearch.com (K.P.)

**Keywords:** sensor tag, flexible tag, near-field communication, packaging, temperature, ethylene, printed electronics, resistive sensor, capacitive sensor, PET

## Abstract

Wireless sensor tags in flexible formats have numerous applications; some are commercially available for specific target applications. However, most of these wireless sensor tags have been used for single-sensing applications. In this study, we designed a printed circuit board (PCB) module (13 mm × 13 mm) for near-field communication-enabled sensor tags with both electrical resistance and capacitance read-out channels that enables dual-channel sensing. As part of the wireless sensor tag, a square antenna pattern was printed directly on a flexible poly(ethylene terephthalate) (PET) substrate and integrated into the PCB module to demonstrate a dual-channel temperature and ethylene gas sensor. The temperature and ethylene sensors were printed using a positive temperature coefficient ink and a tin oxide (SnO_2_) nanoparticle ink, respectively. With dual sensing capabilities, this type of sensor tag can be used in smart packaging for the quality monitoring of fresh produce (e.g., bananas) by tracking temperature and ethylene concentration in the storage/transport environment.

## 1. Introduction

Near-field communication (NFC) is a low-power wireless technology operating within short distances (usually 40 mm or less) in a frequency range centred on 13.56 MHz [1]. One key feature of the NFC platform is the feasibility of passive devices powered by separate energy sources, usually NFC readers. These are often referred to as ‘battery-free’ or ‘battery-less’, differentiating NFC from other wireless platforms, such as Bluetooth and Wi-Fi, which require batteries or other power sources [2,3,4,5,6,7,8,9,10]. Since most modern smartphones are equipped with an NFC function, this technology has become an integral part of our daily lives. Applications include contactless EFTPOS (electronic funds transfer at point of sale), accessing information, exchanging contact details, high-volume rapid processable tickets for events and transportation, secure access and identity cards, asset tracking tags, toys, and games. NFC-based sensors are also becoming more versatile and widespread in applications in the biomedical space, health care, and food quality monitoring [1]. NFC-enabled smart sensor tags or ‘smart patches’ focusing on human health monitoring have been developed. Examples include epidermal patches with strain or temperature sensing [11,12,13,14], full-body sensor patches for pressure and temperature mapping [8], body sensor networks on clothing [4], pulse oximeters on fingernails [9], optical skin characterisation [10], and sweat monitoring and analysis [6,7,15].

NFC sensors for fresh food quality and safety monitoring have also been demonstrated in the literature [2,3,16,17]. A light-energy-harvesting NFC patch for temperature sensing during cold food storage has been developed. Still, most of its major components (e.g., temperature sensor, NFC antenna, and solar cells) were not fully integrated into the flexible patch but just connected to it via hard wires [16]. A flexible NFC electronic system has also been demonstrated, with the antenna being part of the patch. However, the sensors (a temperature sensing integrated circuit (I.C.), a load cell for weight measurement, and a pH meter electrode) were connected to the antenna separately by soldering [3]. Al-though a rigid NFC device for fruit moisture loss control was suggested, which operated by monitoring weight changes [2], to the best of the authors’ knowledge, research on multifunctional sensing-focused NFC sensors in an integrated and flexible format (i.e., every component on one flexible substrate) has not been conducted to meet the sensing requirement in real-life situations.

Ethylene is a colourless, sweet-smelling gas released from fruits as they ripen. It is a volatile compound that can be monitored to reduce or prevent food spoilage during various supply chain and storage stages. This is especially relevant in postharvest handling and the subsequent supply chain stages [18,19]. According to the U.S. Department of Agriculture, it is estimated that U.S. supermarkets lose about 11.4% of fresh fruit and 9.7% of fresh vegetables due to spoilage every year [20], and one significant reason for spoilage at the retail level is uncontrolled exposure to ethylene. Ethylene gas detectors are already commercially available but are usually bulky and expensive, require frequent calibrations, and have a limited lifetime (usually 2–3 years). Instead, resistive and capacitive sensors can be compactly integrated into electronics and fabricated on two-dimensional surfaces, resulting in very flat, thin, and lightweight devices that can be easily affixed to the surface of packaging materials such as cardboard. Therefore, numerous research groups have studied electrical signal-based (chemiresistive or chemicapacitive) ethylene sensing [21,22,23,24,25]. For example, a chemiresistive ethylene sensor with a catalytic overlayer of nanostructured tin oxide (SnO_2_) was demonstrated at elevated operating temperatures (350–450 °C) [21]. Ishihara et al. reported a chemiresistive sensor array based on a cascade reaction (ethylene → acetaldehyde → hydrochloric acid) using acid-doped carbon nanotubes [22]. A chemicapacitive sensor using SnO_2_ nanoparticles has also been studied for the detection of ethylene gas [25].

In addition, temperature is another critical environmental factor that affects product deterioration rates and postharvest lifespans of fresh horticultural products, such as fruit and vegetables. For example, up to 20% of all perishable foods may be lost due to a lack of refrigeration infrastructure or access to energy [26]. Therefore, temperature monitoring during product distribution will also ensure the appropriate maintenance of cold chain conditions and reduce power consumption [27,28,29].

While many wireless sensor devices have been reported, the majority of them are limited to detecting one single analyte or parameter. Here, we present an NFC-enabled dual-sensing flexible sensor tag on a flexible packaging substrate with both electrical resistance and capacitance read-outs. As a proof-of-concept, a resistive temperature sensor and a capacitive ethylene gas sensor were successfully fabricated by printing onto the flexible sensing platform directly. Finally, NFC-enabled temperature and ethylene sensing was demonstrated using this dual-sensing device.

## 2. Materials and Methods

### 2.1. Materials

A nano-silver ink (PSI-211, 44% silver loading, Novacentrix, Austin, TX, USA) and a clear thermally stable dielectric epoxy ink (Product No. 118-12, Creative Materials, Ayer, MA, USA, thermal stability—good to +280 °C) were used as conductive and dielectric inks, respectively, for screen printing on standard heat-stabilised flexible poly(ethylene terephthalate) (PET) films (OfficeMax New Zealand, Auckland, New Zealand). SnO_2_ nanoparticle ink (2.5 wt%, Avantama^®^ N-31, Product No. 9076, Avantama AG, Stäfa, Switzerland), cellulose acetate (average Mn ~30,000 by Gel Permeation Chromatography, Product No. 180955, Sigma-Aldrich, Burlington, MA, USA), and tetrahydrofuran (anhydrous, >99.9%, Product No. 401757, Sigma-Aldrich, Burlington, MA, USA) were used as received with no further treatments. Loctite^®^ ECI8120 (solid content 46%, Henkel, Düsseldorf, Germany) ink was used as the positive temperature coefficient (PTC) temperature sensing material, and the resistance was modified by mixing it with the clear epoxy ink. An electrically conductive silver epoxy adhesive (8330S, M.G. Chemicals, Surrey, BC, Canada) was used to integrate the printed circuit board (PCB) onto the PET substrate.

### 2.2. Device Fabrication

A square NFC antenna pattern (70 mm × 70 mm, 1 mm width, 1 mm gap, and four loops) with interdigitated electrodes (IDEs) was screen-printed on a PET substrate (100 µm thick) using nano-silver ink. The stenciled mesh (120T (threads per centimetre) mesh size, polyester mono-filament yarn) was fabricated using the Fotecoat 1850 emulsion. The NFC antenna pattern was designed using the NFC Antenna Design Tool distributed by STMicroelectronics (https://eds.st.com/antenna/#/, lastly accessed on 28 July 2023). The printed pattern was then thermally cured for 10 min at 120 °C in a convection oven. Next, the temperature sensing ink, a 50/50 wt/wt mixture of Loctite^®^ ECI8120 and a clear epoxy ink, was screen-printed on the IDEs and thermally cured for 10 min at 120 °C.

**Figure 1:**
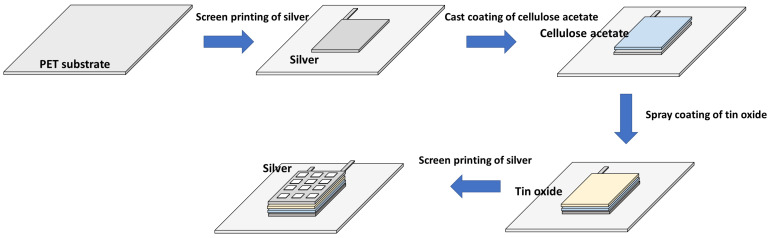
Schematic of the ethylene sensor fabrication process: (1) screen printing a rectangular silver electrode on a PET substrate, (2) coating a solution-cast cellulose acetate layer onto the silver electrode, (3) depositing a spray-coated tin oxide nanoparticle layer onto the cellulose acetate layer, and (4) screen printing a rectangular grid-shaped silver electrode onto the tin oxide layer.

To fabricate an ethylene-sensing chemicapacitor, a four-layer structure was printed on the PET substrate (Figure 1). The four-layer system, including cellulose acetate insulating and SnO_2_ dielectric layers, was suggested by Agarwal et al. [25]. First, a silver layer was screen-printed and thermally cured in the same manner (for 10 min at 120 °C) as the silver antenna. Then, a cellulose acetate layer was solution cast coated using a cellulose acetate solution (10 wt%) in tetrahydrofuran (THF) at room temperature, followed by drying at room temperature for 24 h under ambient conditions. The layer of cellulose acetate was used to prevent a through-plan short-circuit [25]. Next, a tin oxide (SnO_2_) nanoparticle layer was spray-coated (25 psi pressure and 8 L/min air flow rate) on top of the cellulose acetate layer using a mask with a rectangular opening and dried at room temperature overnight. Finally, a grid mesh-shaped silver layer was screen-printed on top and cured under the same conditions as the other silver layers.

The in-house designed PCB with an integrated circuit (IC), NXP^®^ NHS3152 (NXP, Eindhoven, The Netherlands) was connected to the two endpoints of the antenna and two IDEs, respectively, using an electrically conductive silver epoxy adhesive.

### 2.3. Characterisation

The surface morphologies of the screen-printed and thermally cured silver antenna pattern, the temperature sensing layer, film-cast cellulose acetate, and spray-coated SnO_2_ layers were examined by a field-emission scanning electron microscope (FE-SEM, JSM-6700F, JEOL, Akishima, Japan) at a voltage of 3 kV. The cross-sectional morphologies of the final temperature sensor and ethylene sensor were further characterised by FE-SEM. Thin strips (ca. 2 mm wide) were cut from the sample using razor blades, then partially cut on the back side (substrate side). Then they were immersed in liquid nitrogen and broken while frozen, using tweezers. The samples were sputter-coated with chromium (10 nm) before loading. From the SEM images, more than 50 particles of silver and SnO_2_, and cellulose acetate film grains were randomly selected for the size distribution, and their particle/grain diameters were measured using ImageJ software (National Institutes of Health, Bethesda, MD, USA).

### 2.4. Sensors Testing

The temperature sensor testing was conducted in the temperature range between 20 and 50 °C in a convection oven. The electrical resistance read-out of the NFC tag was compared with the read-out of a Type K thermocouple by using a multimeter (FLUKE 233, Fluke, Everett, WA, USA). The ethylene sensor testing was conducted in a 520 mL polypropylene chamber. The sensor was located in the chamber and known volumes of ethylene gas (at atmospheric pressure) were taken from a compressed gas cylinder and injected through a rubber septum to create different ethylene gas concentrations (100, 250, and 500 ppm). The capacitance changes were recorded by an LCR meter (Keysight U1733C, Keysight Technologies, Santa Rosa, CA, USA).

## 3. Results and Discussion

**Figure 2:**
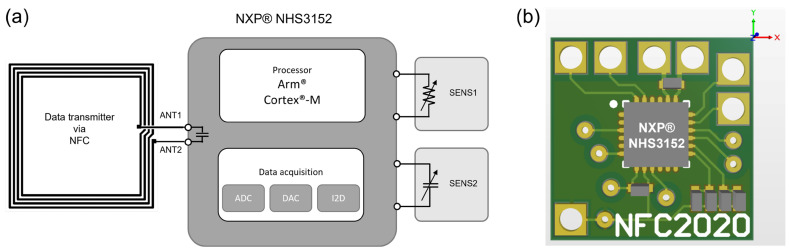
(**a**) A simplified diagram of the suggested dual-sensing NFC platform and (**b**) a schematic of the PCB design (13 mm × 13 mm). Note that SENS1 represents a resistive sensing channel, while SENS2 represents a capacitive sensing channel. In this context, ADC refers to an analogue-to-digital converter, DAC stands for a digital-to-analogue converter, and I2D represents a current-to-digital converter.

**Figure 3:**
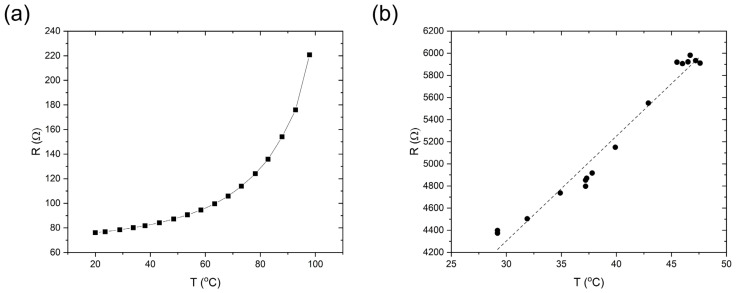
The temperature sensing response of (**a**) the pristine Loctite^®^ ECI8120 ink and (**b**) a 50/50 wt/wt mixture of Loctite^®^ ECI8120 and a clear epoxy ink screen-printed onto the IDEs.

**Figure 4:**
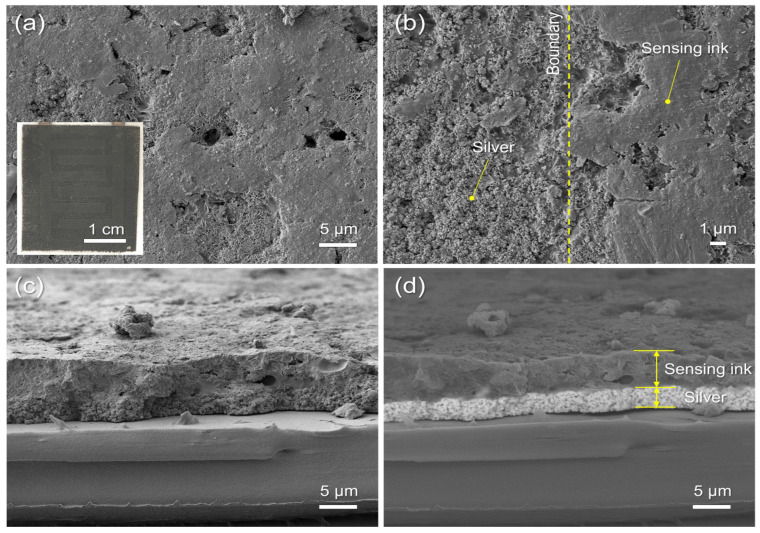
SEM images (top-view) of screen-printed temperature sensor: (**a**) temperature sensing layer (a 50/50 wt/wt mixture of Loctite^®^ ECI8120 and a clear epoxy ink) deposited on silver IDEs, and (**b**) the edge of the printed sensing layer showing the bare silver on the left, and SEM images (cross-section) of screen-printed temperature sensor: (**c**) lower secondary electron detector mode and (**d**) backscattered electron detector mode, at ×2000 magnification for (**a**,**c**,**d**) and ×5000 magnification for (**b**). The inset of (**a**) is a photograph of a printed temperature sensor (23 mm × 19 mm) on silver IDEs. Note that the backscattered electron detector mode provided clear differentiation between the temperature sensing layer and the silver layer, enabling accurate estimation of the thickness of both layers.

The NXP^®^ NHS3152 is the integrated circuit (IC) used for the dual-sensing NFC sensing tag. The IC uses an ARM^®^ Cortex^®^-M processor and a data acquisition unit for resistance and capacitance channels, with NFC/RFID connectivity (Figure 2). The designed PCB with six contact pads was compact enough (13 mm × 13 mm) to be mounted on a flexible substrate with an electrically conductive silver epoxy adhesive. A screen-printable PTC ink was used to design flexible temperature-sensing elements. PTC inks are usually carbon-based inks with electrical resistive properties that increase with temperature. There have been studies on using PTC inks for printable and flexible temperature sensors [30,31]. These have highlighted aspects of large-area temperature sensors and the facile printability of the inks compared to other types of temperature sensors. When the sensing layer was printed with pristine commercial screen-printed PTC ink (24 mm × 18 mm), we found that the electrical resistance of the sensing layer changed from 70 to 200 Ω as the temperature increased from 20 to 100 °C (Figure 3a). A linear relationship between temperature and resistance was observed from 20 to 50 °C; however, when the temperature increased above 50 °C, the resistance increased rapidly, with the thermal coefficient no longer a constant. This resistance range achieved with the pristine commercial PTC ink was outside the operating limits of the commercial PCB (1 kΩ and above) (Figure 3a). Therefore, the PTC ink was mixed with a commercial thermally stable dielectric epoxy ink at 50/50 wt/wt to increase the resistance of the printed sensor to above 1 kΩ. When this formulation was screen printed on top of the IDEs, the resistance was in the range of 4 to 6 kΩ as the temperature increased from 30 to 50 °C, which is in the linear region (Figure 3b). SEM images (both top and cross-sectional views) of the screen-printed temperature sensor are shown in Figure 4. Uniform coverage of the sensing layer on the silver electrodes suggested homogeneous mixing of the PTC ink and dielectric epoxy ink (inset of Figure 4a). Figure 4b shows the edge of the printed sensing layer with the bare silver area on the left and the sensing ink-covered area on the right. The backscattered cross-sectional image (Figure 4d) also shows the boundary between the silver IDEs and the temperature-sensing layer (approximately 5 μm thick). It confirms the excellent adhesion of both layers with no sign of delamination.

**Figure 5:**
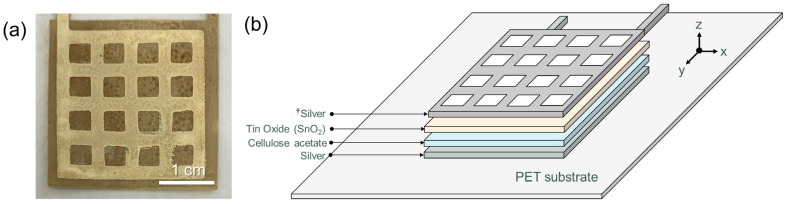
The fabricated capacitive sensor on flexible PET substrate: (**a**) a photograph of a multi-layer printed ethylene gas sensor and (**b**) a schematic of the multi-layer ethylene sensor structure (PET/silver/cellulose acetate/tin oxide/silver). ^†^ The area of the upper silver layer is slightly smaller than the other three layers to prevent any potential short-circuit.

Unlike the single-layer screen-printed resistive temperature sensor, the chemicapacitive sensor was fabricated with multi-layer processing [25]. A photograph of the fabricated chemicapacitive sensor is shown in Figure 5a, and its multi-layer structure is illustrated in Figure 5b. First, the bottom electrode was screen-printed in a square pattern (30 mm × 30 mm) on PET using nano-silver ink. Then, cellulose acetate was coated onto a silver pattern of the same size as the bottom electrode. A defect-free cellulose acetate film was essential for successful device operation. Otherwise, it was prone to short-circuiting between the two silver electrodes after device completion. A thicker cellulose acetate layer significantly reduced the risk of device failure. However, it is noted that the thickness of the cellulose acetate layer also determines the capacitance range of the chemi-capacitive sensor, which is why care must be taken when optimising the layer thickness (approximately 10 μm). A cellulose acetate layer of less than 5 μm thick led to frequent short-circuiting, while an approximately 20–50 μm thick cellulose acetate layer showed no sensitivity to ethylene at all. Then, SnO_2_ nanoparticle ink was printed on top of the cellulose acetate layer to construct an ethylene sensing channel [25]. On top of the SnO_2_ layer, the second silver electrode layer was deposited to complete the capacitor in a grid shape to allow gas molecules access to the SnO_2_ nanoparticle surfaces. The capacitance channel available from the PCB we used was limited to a narrow range (4 nF–1.1 µF). To match the capacitance range of our chemicapacitor, the thicknesses of the cellulose acetate and SnO_2_ nanoparticle layers were carefully optimised. Approximately 1 µm SnO_2_ and 10 µm cellulose acetate turned out to be the best compromise between correct capacitance, suppression of short short-circuiting, and sensitivity and allowed the manufacturing of a chemicapacitor that was readable by the chip we used.

**Figure 6:**
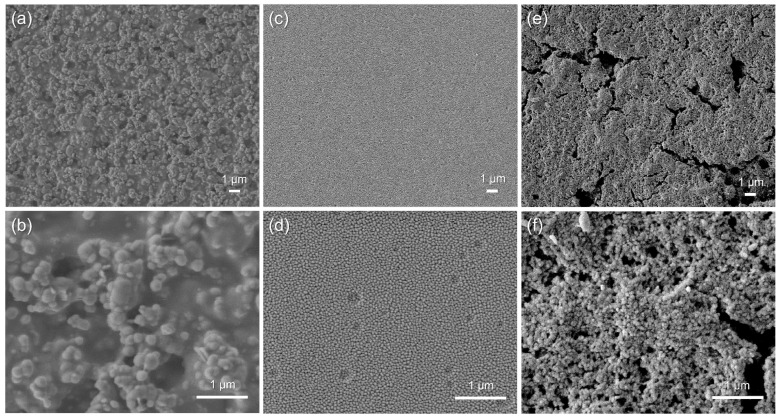
SEM images (top-view) of each layer of the capacitive ethylene sensor: (**a**,**b**) screen-printed and thermally cured silver, (**c**,**d**) film-cast cellulose acetate, and (**e**,**f**) deposited SnO_2_ nanoparticles at two different magnifications (×5000 and ×20,000).

**Figure 7:**
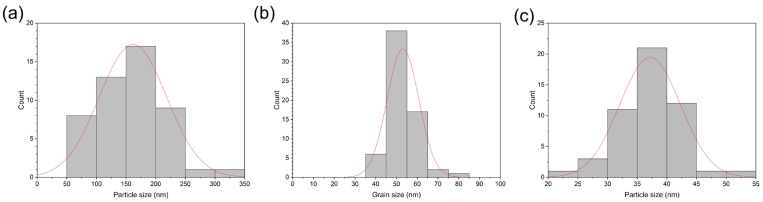
Size distributions of (**a**) conductive silver ink particle after annealing (average particle size of 162 nm), (**b**) cellulose acetate film grains (average grain size of 53 nm), and (**c**) the deposited SnO_2_ nanoparticles (average particle size of 37 nm) as part of the multi-layer chemicapacitor.

**Figure 8:**
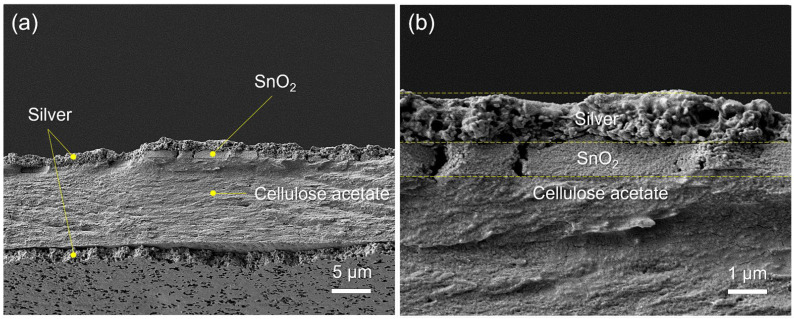
SEM images of (**a**) a cross-sectional view of the capacitive ethylene sensor construction (silver/cellulose acetate/SnO_2_/silver) at ×2000 magnification and (**b**) its top part at ×10,000 magnification. Note that the PET substrate was excluded in these SEM images due to delamination of the functional stack during sample preparation.

Cracks or other surface defects can result in short-circuiting or other types of failure of this multi-layer chemicapacitor sensor. SEM was therefore used to analyse the surface and cross-sectional micromorphology of the chemicapacitor. Surface-view SEM images were recorded of each layer of the chemicapacitor at two magnifications (×5000 and ×20,000) (Figure 6). The nano-silver inks were still in a particulate form, but the higher-magnified image showed evidence of thermal sintering–inter-connected particulates (Figure 6a,b). The cast film of cellulose acetate on the silver electrode has a smooth surface with no cracks or defects (Figure 6c). The higher-magnified image also shows craters less than 200 nm in diameter on the surface, which are expected to be present only on the surface due to solvent evaporation (Figure 6d), which is frequently observed in solution cast films. Note that after solution casting, it was dried at room temperature under ambient conditions to slow down solvent evaporation. However, when heated, solvent evaporation was accelerated, which can lead to the formation of cracks or larger craters. The SnO_2_ nanoparticle ink was spray-coated on top of the cellulose acetate layer and subsequently heated, and the nanoparticulate morphology (with an average particulate diameter of 37.2 nm) was still maintained after the deposition and thermal treatment processes (Figure 6e,f). Figure 7 shows the size distribution of the annealed conductive silver particles (Figure 7a) with an average particle size of 162 nm, the cellulose acetate film (Figure 7b) with an average grain size of 53 nm, and the deposited SnO_2_ nanoparticles, which displays a standard distribution curve with a grain size range between approximately 20 nm and 55 nm (Figure 7c). Cross-sectional images of the multi-layer chemicapacitor device were also recorded by SEM (Figure 8). Upon sample preparation, the device stack delaminated in its entirety from the PET substrate, which is why the latter is not visible. The boundaries between the various layers can be clearly observed, and the thickness of each layer is approximately 1 μm, 1 μm, and 10 μm for silver, SnO_2_ nanoparticles, and cellulose acetate, respectively. The cellulose acetate layer is densely packed and non-porous, so electrical short-circuiting in the thickness direction was prevented.

**Figure 9:**
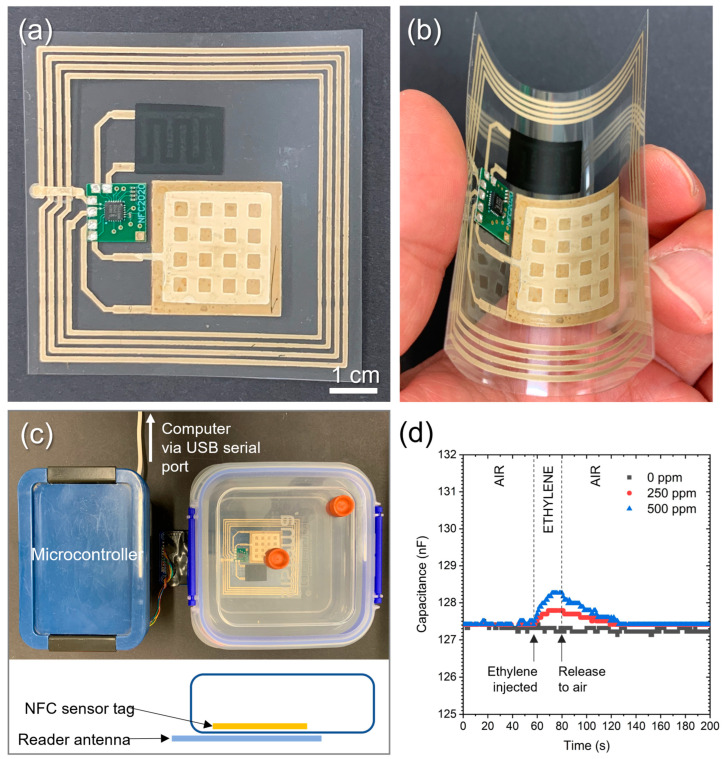
Dual-sensing NFC tag: (**a**) a photograph of the sensor tag, (**b**) its demonstrated flexibility, (**c**) the NFC sensor tag testing experimental setup, and (**d**) the ethylene sensing response of the dual-sensing NFC tag.

The fabricated flexible NFC sensor tag on the PET substrate is shown in Figure 9. The bendability of the tag is also demonstrated (Figure 9b). The temperature and ethylene sensors were fabricated with the PTC ink and SnO_2_ nanoparticle ink, as explained earlier. Both sensors were in the two-dimensional planar design, allowing large sensing areas. The functionality of the temperature sensor part has already been demonstrated above (see Figure 3). Because the read-out of both devices occurs via two independent channels, they can both be operated at the same time, allowing simultaneous monitoring of both target values. The ethylene sensing behaviour was tested with the sensor tag in an ethylene chamber and the NFC reader outside the chamber, as demonstrated in Figure 9c. We observed that the signal increased for 20 s after the gas injections, and it took 50 s for the signal to decrease to the baseline value when the sensors were exposed again to ambient air. The peak capacitances were 128.3 and 127.8 nF when ethylene was injected to reach 500 and 250 ppm concentrations, while the baseline was at 127.2 ± 0.1 nF. These results show that 250 ppm was the minimum concentration of ethylene that our sensor device detected. No changes in capacitance from the baseline were observed in the 100 ppm ethylene environment. Each step of the capacitance read-out values was 0.1 nF, and the demonstrated ethylene sensitivity was not high. Further improvement in the active material (i.e., SnO_2_ nanoparticles) by catalytic doping [25] could enhance the ethylene sensitivity. Reducing the thickness of the cellulose acetate is most likely an alternative option to increase sensing performance, as long as the formation of electrical short circuits between the silver electrodes can be successfully prevented. It is also expected that the signal resolution (currently 0.1 nF) could be improved by modifying the capacitance signal processing circuit of the PCB.

Cross-talk between the two sensors is expected to be an issue that future work should address. While it is highly likely that the capacities of the ethylene sensor will also be influenced by temperature changes, it is expected that the presence of ethylene gas will have negligible, if any, influence on the conductivity of the carbon ink. In this way, the readings from the temperature sensor can be used to correct the results from the ethylene sensor if a proper calibration for its temperature dependence has been carried out before.

## 4. Conclusions

We have successfully developed a dual-sensing NFC sensor tag by incorporating the NXP^®^ NHS3152 chip onto a PCB, along with printed sensors and an NFC antenna, all integrated onto a flexible PET substrate. The temperature sensing capability was achieved through the use of a resistive temperature sensor printed with commercial PTC ink. In addition, an ethylene chemicapacitive sensor was fabricated with a multi-layer structure. The temperature and ethylene dual wireless sensing capabilities were demonstrated with an NFC reader connected to a computer. The results also revealed some limitations of the sensing capabilities with the current experimental design (e.g., narrow range of signals and low sensitivity). To address these limitations and enhance the performance of both the resistive and capacitive sensors, further development of the sensing material and/or the sensor design is necessary. With such advancements, it is expected that this dual-sensing NFC tag could provide a wireless sensor platform for various other types of chemiresistive and chemicapacitive sensors directly printed and fabricated on a planar tag.

## Data Availability

The data presented in this study are available on request from the corresponding authors.
